# Whole genome sequencing in (recurrent) glioblastoma: challenges related to informed consent procedures and data sharing

**DOI:** 10.1007/s00701-024-06158-z

**Published:** 2024-06-14

**Authors:** Mira C. Hasner, Mark P. van Opijnen, Filip Y. F. de Vos, Edwin Cuppen, Marike L. D. Broekman

**Affiliations:** 1 Department of Neurosurgery, Haaglanden Medical Center, The Hague, The Netherlands; 2 Department of Cell and Chemical Biology, Leiden University Medical Center, Albinusdreef 2, Leiden, 2333 ZA The Netherlands; 3 Department of Medical Oncology, Utrecht University Medical Center, Utrecht, The Netherlands; 4 Hartwig Medical Foundation, Amsterdam, The Netherlands; 5 Center for Molecular Medicine and Oncode Institute, University Medical Center Utrecht, Utrecht, The Netherlands; 6 Department of Neurosurgery, Leiden University Medical Center, Leiden, The Netherlands

**Keywords:** Whole genome sequencing, Recurrent glioblastoma, Cognitive impairment, Informed consent, Data sharing

## Abstract

Increased use of whole genome sequencing (WGS) in neuro-oncology for diagnostics and research purposes necessitates a renewed conversation about informed consent procedures and governance structures for sharing personal health data. There is currently no consensus on how to obtain informed consent for WGS in this population. In this narrative review, we analyze the formats and contents of frameworks suggested in literature for WGS in oncology and assess their benefits and limitations. We discuss applicability, specific challenges, and legal context for patients with (recurrent) glioblastoma. This population is characterized by the rarity of the disease, extremely limited prognosis, and the correlation of the stage of the disease with cognitive abilities. Since this has implications for the informed consent procedure for WGS, we suggest that the content of informed consent should be tailor-made for (recurrent) glioblastoma patients.

## Introduction

The understanding of tumor genesis and -progression is improving due to the combined use of advanced data analysis techniques with next generation sequencing (NGS) and whole genome sequencing (WGS) [3]. Results may facilitate personalized medicine through the identification of therapeutically relevant alterations and pharmacogenetics, realizing the assessment of genomic variants impacting therapeutic potential or side-effects [19]. Simultaneous development of targeted therapies steadily increases the relevance of genomic essays in clinical cancer care of patients with solid tumors [16]. However, the use of WGS and subsequent targeted therapies is not (yet) standard-of-care for patients with tumors of the central nervous system [8]. Various papers have described the genomic landscape of glioblastoma [7, 12, 27], the most common primary malignant brain tumor. Currently, NGS is used for diagnosis and identification of molecular alterations with potential therapeutical implications in glioblastoma [8]. The benefit of routine application of WGS for patients with recurrent glioblastoma is currently being explored in a prospective clinical trial [43]. WGS provides a wealth of information that could contribute to a better understanding of pathogenesis and to the development of novel therapies, therapy monitoring and treatment optimization [25]. Sharing genome-wide genomics data in combination with clinical information with databanks has the potential to improve future care for patients.

Compared to NGS, which uses a predefined gene panel, WGS sequences the whole genome including non-coding areas. Moreover, WGS is a reliable technique for detecting structural variants such as gene fusions. The ‘completeness’ of WGS has the additional potential of minimizing interlaboratory variations in NGS panel composition [44]. WGS reports present extensive data on the genomic alternations in cancer cells, as well as a comprehensive view of normal tissue and tumor clonality. It therewith provides immediate clarity on whether alterations are somatic or germline and could reveal additional (hereditary) information unrelated to the tumor. Such results are called unsolicited findings (UF) and may have practical and ethical consequences that are difficult to predict upfront.

Current European Association of Neuro-Oncology (EANO) guidelines address how and when to test for predictive genetic alterations, how to report findings, and how to attribute pathogenic and clinical relevance [8, 45]. However, there are no international recommendations on the format of informed consent procedures for WGS in neuro-oncology. Insecurities surrounding the sensitivity of genomic data and difficulties in predicting the impact of findings amplify the importance of patient counseling on informed consent procedures for WGS and data sharing. We investigate whether traditional standards of informed consent are clinically feasible in the context of WGS for patients with (recurrent) glioblastoma, who often suffer neurocognitive impairment. We further explore ethical implications described for WGS in oncology; the legal frame of existing models of informed consent; and the role of patient characteristics on their preferences regarding the receiving and sharing of genomic data.

## Methods

### Study purpose and search strategy

In this narrative review, the primary purpose of this study was to examine the current literature on ethical implications related to informed consent procedures and data sharing in the context of WGS in (recurrent) glioblastoma. The PubMed database was used and the search strategy was not restricted to brain tumors or oncology, because of the limited literature available. Therefore, the search strategy was composed of the following keywords:

(“Whole Genome Sequencing“[MeSH] OR “whole genome sequencing” OR “WGS”) AND ((“Informed Consent“[MeSH] OR “informed consent” OR “consent”) OR (“Data Sharing“[MeSH] OR “Data Management“[MeSH] OR “Confidentiality“[MeSH] OR “data sharing” OR “data management” OR “data privacy”) OR (“Ethics“[MeSH] OR “Bioethical Issues“[MeSH] OR “ethical implications” OR “ethical considerations” OR “bioethics”) OR (“Legislation as Topic“[MeSH] OR “Jurisprudence“[MeSH] OR “legal implications” OR “legal considerations” OR “law” OR “regulations”)).

Articles of potential interest were screened for their relevance based on the following criteria. First, they should address either one or more of the next topics related to the use of WGS in humans: ethical implications, legal implications, issues regarding informed consent, issues regarding data sharing. Second, articles focusing solely on technical aspects of WGS without discussing ethical implications were not included. Finally, articles not written in English were excluded.

### Data extraction and analysis

Data extraction focused on key themes related to ethical considerations, including (1) patient autonomy and informed consent, (2) privacy and data sharing practices, (3) legal frameworks and regulations, and (4) broader bioethical discussions to WGS in oncology. Subsequently, the ethical implications of WGS were explored by synthesizing data on currently described issues with the use of WGS in oncology, ethical principles of autonomy in the context of participant comprehension and data sharing, and ethical dilemmas arising from the potential for incidental findings, genetic privacy concerns and implications for family members. To explore the legal frameworks governing WGS, data was synthesized on relevant legal precedents and case law, and international policies on data sharing, storage and protection on the context of genetic information.

The results were synthesized to provide a comprehensive overview of the ethical and legal implications of WGS in (recurrent) glioblastoma. Themes were organized into sections covering informed consent, data sharing, privacy concerns, and legal considerations. To assess whether traditional standards of informed consent are clinically feasible in the context of WGS, this study included a focused examination of articles discussing limitations and benefits of different models of informed consent procedures as used in medical research as well.

## Results

### Ethical implications of informed consent for WGS in oncology

WGS analysis could result in the disclosure of sensitive information, which may have (psychological) consequences for patients and their relatives. Informed consent procedures can significantly endorse patient autonomy and should carefully be considered. Factors that affect informed consent procedures for WGS analysis and data sharing include privacy concerns and preconditions for autonomy, such as information disclosure and participant comprehension [41]. 

### Information disclosure and relevance of findings

Unclear relevance of findings makes disclosure about potential risks and consequences of WGS challenging and could result in misguided perceptions of beneficence and harm [22]. Genomic alterations may have a different clinical relevance across cancer types and the evidence of actionability can range from hypothetical target for treatment to established therapeutic efficacy [8]. Clinical relevance of findings is based on their predictive value in relation to disease progression, the probability of treatment response, actionability in terms of consequential interventions and whether there are immediate consequences for patients. Guidelines provided by the American College of Medical Genetics and Genomics (ACMG) [37] and joint recommendations of Clinical Genome Resource (ClinGen), Cancer Genomics Consortium (CGC) and Variant Interpretation for Cancer Consortium (VICC) [23] can be used to classify pathogenicity of germline and somatic variants, respectively. Following, there are scoring systems that assess the levels of evidence supporting the clinical value of pathogenic variants as targets for treatment. The European Society for Medical Oncology (ESMO) Scale for Clinical Application of molecular Targets (ESCAT-classification system) [30], OncoKB [10] and CIViC [21] assess the degree of actionability of somatic variants, while ClinVar [26] provides interpretations of germline variants. However, the majority of genomic data available in databases used to assess biological significance of variants include only information on non-central nervous system tumor types. The value of these scales is dependent on international differences in regulatory approval of drugs and the availability of trials [32]. Ideally, an interdisciplinary tumor board discusses the degree of actionability of variants per case.

There is no international consensus about a specific list of genomic variants that should be communicated back to patients [6, 48]. Nor is it obligatory to communicate back any genomic UFs in the European Union (EU). Yet, the ACMG recommends that clinicians should report back genomic variants that are actionable or have phenotypes that are highly penetrant, disease causing or of other medical relevance [33]. Reporting back a default list of findings may violate the ethical norm of ‘the right not to know’ [15]. The question should be raised whether potential clinical benefit and the clinicians’ duty to prevent harm supersedes the principle of autonomy of the patient. Dutch guidelines advice against reporting back genomic findings to patients who have expressed their unwillingness to receive them during informed consent procedures [38]. 

### Participant comprehension

The complexity of genomic concepts may hamper patient comprehension during counseling for WGS analysis, which challenges clinicians to review if autonomous decision-making has taken place. A quantitative multicenter study found that patients who declared to have sufficient knowledge and experience with genomic testing, changed their consent after watching educational videos on receiving information about UFs [4]. Moreover, a survey of patients with refractory, metastatic cancer undergoing WGS analysis further found that their expectations regarding direct benefits of study participation are largely unfulfilled [39]. Despite contrary clinical counseling, the survey concluded that patients expected written reports of sequencing findings, a greater understanding of the causes of their cancer, results making them eligible for participation in clinical trials and disclosure of UFs.

### Protecting patient autonomy and data sharing

Patients may hesitate to share their genomic data due to concerns about potential misuse. Databanks generally secure the patients’ right to protection of personal data technically and in data licenses. Efforts are made to de-identify data by the replacement of personal details with an automatically generated code and through aggregation of data into big data sets. Nevertheless, genomic sequences are per definition unique to an individual and these measures will therefore never eliminate the theoretical possibility of patient reidentification. Regardless, legislation mandates only that sufficient measures need to be taken to ensure reidentification is not possible with reasonable efforts [35]. The sensitivity of data depends on context and its relation to other information, patient interests, and consequential decision-making.

Different countries often have different data protection regulation, which makes sharing data in international research teams challenging. Concerns about unwarranted disclosure of genetic information extend the patient-physician relationship and is further influenced by societal factors, encompassing politics, law, and health care. In general, a higher data protection standard can be expected when there are more institutional and political safeguards in place [46]. An example would be the protection of genetic information in France and Canada, where findings are exclusively allowed to be used for medical and scientific purposes. In the EU discrimination based on genetics is forbidden by law and genetic data is classified as sensitive data under the General Data Protection Regulation (GDPR) [35]. In stark contrast, in the United Kingdom, findings can be used to determine insurance thresholds if policy exceeds a certain financial limit, while in the United States (US) patients may need to disclose genetic findings for certain kinds of insurance [2, 18]. The Genetic Information Nondiscrimination Act (GINA) in the US, which excludes employers with under 15 employees, does not protect against genetic discrimination by disability-, long term care- and life insurance [11]. Accordingly, patients undergoing germline testing have reported fear for discrimination based on genetics, for example by insurance companies or employers [18]. Regulations protecting personal data and conditions allowing for secondary use differ regionally in both the US [20] and the EU [24]. This complicates data transfers between the US and the EU, despite the US-EU privacy shield [5]. Moreover, notwithstanding the necessity of these regulations, they may impact the feasibility of clinical research in which sharing genomics and proteomics data is crucial [13]. 

### Models of informed consent

**Fig. 1:**
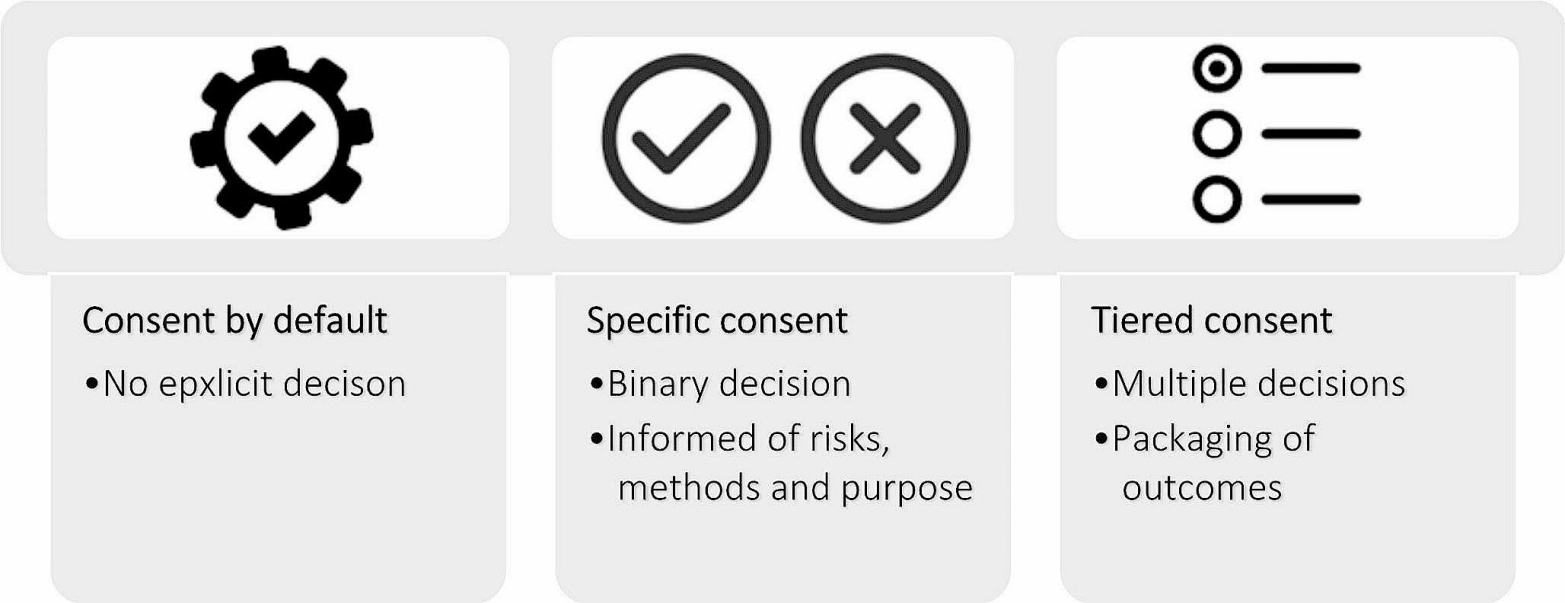
Overview of the types and characteristics of informed consent

Under the influence of legislation different models of informed consent were developed for consent to treatment, research, disclosure of genomic results and data storage [9]. Examples of models available for consideration are *consent by default*, *specific consent*, *tiered consent*, and *broad consent* [46]. Each model is characterized by its own legal context, advantages, and limitations (Fig. 1).

### Consent by default

*Consent by default* is applied when in consenting to participation in a study, patients automatically consent to publicly sharing the results and data of that study [31]. Although this option could limit the administrative burden on researchers, *consent by default* is not legally valid for sharing personal data under the GDPR [35]. Permission for the processing of personal data in the context of providing medical treatment is not necessary. However, explicit consent must be obtained if healthcare providers wish to lawfully use the genetic data for further processing, such as research.

### Specific informed consent

*Specific informed consent* refers to the binary decision of a patient after being informed of potential risks, the methods and purpose of a study or treatment. The *specific informed consent* that is given in daily clinical care or medical randomized controlled trials is not sufficient to handle large scale genomic data, because the clinical relevance of the wide range of possible- and potentially UFs in WGS is not apparent in advance and can be difficult to express in terms of risk or consequences. However, recital 33 of the GDPR recognizes that it is not always possible to describe the purpose of research at the moment of data collection [35]. Specific informed consent could be used to offer patients who consented to WGS analysis the option of *opting out* on receiving any genetic information, in protection of their right not to know.

### Tiered consent

In solution to overwhelming patients with excessive amounts of information and limiting the administrative burden imposed on researchers, *tiered consent* wields a binning approach. Patients are presented with categorized packages to which they can choose to *opt in*. Table 1 depicts an example of pre-arranged packages of results based on relevance [4]. 

**Table 1:** Example of pre-arranged packages of results used in tiered consent

Categories of unsollicited findings	
Actionable	Findings regarding a genetic predisposition for disease with available treatment or prevention.
Non-actionable	Findings regarding a genetic predisposition for disease for which no effective treatment or prevention has been established yet.
Heritable	Findings regarding a genetic predisposition with reproductive relevance and relevance to relatives. These findings do not necessarily have direct consequences for the patient.
Unknown relevance	Findings with no known genetic or clinical relevance.

Organizations and studies have made recommendations for returning genomic findings in oncology. An example of current practice would be the combination of *specific informed consent* and *tiered consent* [28], corresponding with recent suggestions by the Dutch guideline on molecular tumor diagnostics [38]. Primarily, patients should be offered the option to *opt out* of the disclosure of any genetic information. If they are open to receiving genetic information, a default package of solicited findings that are actionable, valid, and accurate will be disclosed. Subsequently, patients can *opt in* on distinct categories of UFs through the *tiered consent* approach. This opportunity to differentiate between options could improve expectation management in counseling and enhance patient autonomy. Research showed that participants enrolled through *tiered consent* were less likely to change their consent for sharing genetic information post-debrief in comparison to through *consent by default* and *specific consent* [31]. Heedful selection of consent procedures and design of bio-informatic analysis that are selective for specific genomic findings could further provide solutions in the dilemma of selecting which findings to report back to patients.

### Broad consent

In addition to consenting to primary research, patients could be asked to share their genomic data with biobanks or databanks. The Office of Human Research Protections revised the Common Rule in 2018 [36] and effectively introduced a new category of informed consent in January 2019: *broad consent*. This option endeavors to increase transparency with advancing technology and big data, where personally identifiable data is accumulated into databanks and biobanks [17]. Widespread participation and accumulation of genomic data sets may give rise to global research networks, sequence reference libraries and connectivity between scientists and their discoveries. To maximize public profit, health data should be made findable, accessible, interoperable, and reusable, conform the FAIR-principles [47]. Factors complicating *broad consent* are the limited control over unspecified future use of data, indefinite storage and use of material and the limited ability for participants to withdraw. It could be argued that patient interests are not thoroughly being safeguarded by consent at the moment of data collection [5]. 

### Focus points in a population with (recurrent) glioblastoma

Patients with glioblastoma have a very limited prognosis and many patients are suffering cognitive or neurological impairments as a result of treatment or disease related factors [40]. This, combined with the relative rarity of the disease and the lack of standard-of-care in the recurrent setting, makes this patient population different from other patients with cancer and might require a tailored approach to informed consent for WGS. Since the presence of cognitive or neurological impairments may be seen in the primary setting as well, the following considerations apply to both primary and recurrent glioblastoma.

Previously, it has been shown in solid tumor patients that specific patient characteristics and personal context, such as demographics and stage of disease, affect preferences regarding disclosure of genomic findings through *tiered consent* [4]. These characteristics include experienced quality of life, depressive feelings, and having a college degree. Patients with first- and second-degree relatives were more interested in UFs of reproductive relevance. Notably, patients with curative treatment options were less willing to receive UFs in general than advanced care patients. Age, health literacy, experience with tumor profiling, and sociodemographic factors play a crucial role in the decision-making process [4]. These findings demonstrate that next to the potential actionability and clinical relevance of genomic findings, patient characteristics might impact preferences in receiving findings and sharing genomic data.

In relating these characteristics to patients with (recurrent) glioblastoma, it should be noted that there are no curative treatment options for glioblastoma. Determining heredity with germline research does therefore not have consequences in terms of preventive treatment options for relatives. Experienced quality of life and feelings of depression are relevant factors in patients with incurable disease. Clinicians obtaining informed consent should be aware of neurocognitive impairments magnifying the previously described challenges that arise in counseling for WGS. Patient autonomy should be valued and preserved as much as possible. Next to experienced quality of life, the importance attributed to quality of life is an important factor in decision making for (clinicians treating) patients with (recurrent) glioblastoma. This population may need more guidance than other oncological populations. Digital tools, such as educational videos for patients and e-learnings for health care professionals [38], could increase the focus on patient education and improve management of patient expectations.

While WGS reports may identify potentially actionable molecular alterations, there are no registered genotype-phenotype correlations with defined clinical consequences known for glioblastoma and ESCAT-scores are still low. Currently, the number of studies initiated for targeted therapies in the recurrent glioblastoma population is limited. Nevertheless, in case an actionable target is identified, recurrent glioblastoma patients might be offered targeted treatment therapy.

In patients with a limited prognosis, like (recurrent) glioblastoma, managing hopes and expectations is important. Especially since this might affect the information (such as UFs) they would like to receive. Indeed, there is a risk that consent procedures are biased by *therapeutic misconception* or *therapeutic hope* [1]. *Therapeutic misconception* means that patients have a false belief that they will obtain clinical benefit from participating in research. This can be resolved by identifying and correcting the patients’ false beliefs and providing tailored information, but there is no evident solution to *therapeutic hope*, which exists when there is even the slightest chance at benefit for the patient.

Subjecting patients to further interventions, especially an invasive procedure to obtain fresh frozen tumor samples with the sole purpose to perform WGS, is currently not justifiable, because chances at medical benefit are small and the actionability of potential targets is uncertain. It is crucial for clinicians who provide tailored information to be transparent about difficult topics, such as limitations in predicting immediate consequences based on clinical relevance and the lack of evidence for treatments in early experimental phase I trials. The alternative option of best supportive care should be considered.

In addition to patient characteristics and unknown actionability of findings, rarity of disease may play a role in decision making. Patients with (recurrent) glioblastoma, as well as patients with other (rare) diseases, might hope to benefit other patients with the same disease. A survey exploring motives for participation in the LeukoTreat program for genetically inherited neurodegenerative disease showed that patients and their families both hoped that their participation would contribute to a better understanding of the progress and causes of the disease, discoveries with (non-)therapeutic impact and more efficient diagnostic tests [14]. These altruistic motives were also observed in a survey by the Dutch Federation of Cancer Patient Organizations, which showed that most cancer patients agree to secondary use of their personal health data without separate consent [34]. This reveals a compassion for future patients. Rare disease communities have the tendency to be more engaged in comparison to populations with more common diseases.

## Conclusion

NGS and WGS are increasingly being used in neuro-oncology, yet there is no global consensus regarding informed consent for WGS and sharing genomic data in (neuro-)oncology [29]. There are several models available for consideration, of which the benefits, limitations and legal context were discussed. We conclude there are many specific challenges for the population of patients with (recurrent) glioblastoma, related to the rarity of the disease, its’ extremely limited prognosis, and the correlation of the stage of the disease with cognitive abilities. Especially cognitive impairments magnify the challenges that arise during counseling for WGS, such as information disclosure and participant comprehension. From an ethical perspective, it is important to recognize vulnerability in cohorts. This vulnerability, that is not exclusive to recurrent glioblastoma patients, may point to a limited capacity to consent and increased sensitivity towards coercion or exploitation.

We suggest that the content of informed consent should be specific to patient populations. A combined model [38] of specific- and tiered consent was proposed for WGS in (recurrent) glioblastoma. The binning approach used in tiered consent has been demonstrated to enhance patient autonomy and it can be adjusted according to the interests of specific populations. *Broad consent* is suggested in the context of sharing personally identifiable data with databanks, though it raises concerns about patient autonomy. In parallel, development of meta-governance solutions should be prioritized to facilitate widespread use of genomic data and international collaborations [13, 42]. 

Future studies determining the preferences of vulnerable cohorts, such as (recurrent) glioblastoma, could further enhance preservation of autonomy prior to standardization of informed consent procedures. Understanding how patient characteristics influence patient preferences in receiving findings could influence categorization based on relevance in tiered consent. It would be interesting to explore whether patients with a limited prognosis and rarity of disease are more prone to an altruistic approach in comparison to people with common disease. For example, to investigate their interest in possible consequences for relatives or the benefit of the patient population; whether they are more willing to donate their data to databases for research; and whether a limited prognosis of disease influences the fear for genetic discrimination. Determining the preferences of vulnerable cohorts upfront could help patients, physicians, and science.

## Data Availability

Not applicable.
